# Offerings
*Dr. Hodson does not wish to be understood as claiming originality of invention or discovery for any of the "offerings" he presents. They are simply things from which he has derived much benefit in his practice; and he hopes that those unacquainted with any of these items may derive similar benefit from them.—Eds.


**Published:** 1878-08

**Authors:** J. F. P. Hodson

**Affiliations:** New York.


AMERICAN JOURNAL

OF

DENTAL SCIENCE.

Vol. XII. THIRD SERIES—AUGUST, 1878. No. 4.

### ARTICLE I.

### *Offerings.**

BY J. F. P. HODSON, D. D. S., NEW YORK.

1. Bibulous paper cut into long diamonds—say three inches long by three-fourths of an inch wide—and twisted lengthwise into a smooth, hard, double-pointed thread, and kept on hand in quantities, will be found especially adapted, and very excellent, for drying out pulp canals after the bulk of the moisture shall have been removed with the ordinary rolls or pellets; inasmuch as the paper threads will penetrate the canals to their extremities, and *absorb* the contents, without the tendency to pack the latter at the upper end of the root, or push them through the foramen, which cotton wound upon a broach possesses.

---

* Dr. Hodson does not wish to be understood as claiming originality of invention or discovery for any of the "offerings" he presents. They are simply things from which he has derived much benefit in his practice; and he hopes that those unacquainted with any of these items may derive similar benefit from them.—Eds.

2. A pledget of wet spunk in the tweezers will take up the finishings from gold fillings wherever they may have fallen—either upon the teeth, dam, or mucous membrane —and is a good substitute for the spittoon trap for saving gold—the pieces loaded with waste gold being thrown into the vessel of water usually kept for the purpose of rinsing gold from files and burrs, and burned out in the final melting of the total washings.

3. If, after having applied the rubber dam, it be found that the holes have been placed too near together in any place, or any one slightly torn in applying, and that in consequence the moisture exudes about the teeth, a small pledget of the spunk wedged between the teeth at their necks, from both the buccal and lingual surfaces, and so treating one or two spaces on each side of the tooth to be filled, will entirely obviate the difficulty. Even though the pellets do not wedge back the leakage *in toto*, but require changing, even two or three times during the operation, it is unquestionably better to thus compromise with the error of judgment than to subject the patient to an entire reapplication of the dam. It is hardly necessary to observe that this course is not an admissible one, when the exudation comes from the space where the filling is being done.

4. A veritable "friend in need," sometimes, is a little appliance which I may call a "rubber dam stopper," with which an accidental puncture of the rubber may be *quickly* and *certainly* sealed. In the earlier years of the rubber I employed ordinary corks for this purpose; but from the difficulty of keeping them in their place, their liability to suddenly jump out of the opening and flood an operation, and the large amount of room which they absorbed, I some years since substituted these appliances. They are simply disks of any suitable material—hard rubber, soft rubber, cork, etc.—say one fourth to three-eighths of an inch in thickness, with a deep groove cut entirely around their circumference, and equidistant from each face, thus producing, after all edges shall have been nicely rounded, something akin to two round-edged buttons placed face to face, and which may be caught up with the tweezers, and in an instant pushed half way through the puncture, the edges of which close into the deep groove, and so entirely seal the opening. This leaves but a little flat button on either side of the rubber, and consequently involves no loss of room or light, while the groove keeps the appliance perfectly in position. I keep by me half a dozen different sizes, from one-eighth of an inch to one inch in diameter at the bottom of the groove, which variety I have always found sufficient to render instantly harmless any reasonably sized puncture of the dam. They may also be valuably employed in such cases as one's having made a miscalculation in cutting the holes for any particular operation or having torn the rubber in the effort to pass it between teeth, or where one chooses, as many do, to employ the same piece of rubber more than once, by tearing out the isthmus of rubber between the holes, and placing one of these appliances in the single opening thereby produced; after which the rubber may be treated in all respects as a fresh piece. It is seldom, surely, that a skillful operator will need these appliances for punctures; but when they *are* wanted, the need is very sore and urgent.

5. The exquisitely thin sheets of cork which are used by hatters will be found a very convenient form of this material from which to cut small pieces to be placed upon the floors of cavities, as non-conductors of thermal changes.

6. An excellent precaution, in the preparation of pulp cavities, when cutting is to be done for the purpose of enlarging the opening, etc., is to push a little pledget of cotton up or down *each* root beforehand, and beyond where any operating is to be done, to be left there during the operation. It will save the real annoyance of any chips or refuse becoming lodged in the roots, and *assurance* is had of their cleanliness by its withdrawal afterwards.

7. A buffet, made by covering a block—say, 3 or 4 inches long, by $1\frac{1}{2}$ inches wide, and $\frac{1}{2}$ inch thick—with leather, covering this again with chamois, and rubbing a little rouge upon the surface, is convenient to have in the operating case, to polish the burnisher upon *each time* before carrying it to a filling. It will then really accomplish its purpose of *burnishing*, and not drag the surface of a filling, as it would if clogged with gold from a former burnishing.

8. I would suggest to such of my professional brethren as have not already done so, a trial of the compound solution of iodine (commonly called "Lugol's Solution," and the formula for which may be found in any U. S. Pharmacopœia) in most, if not all of the places where tincture of iodine has been the usual remedial agent. It is an aqueous solution instead of an alcoholic one, and as such has, especially when topically employed, greater affinity for the tissues, and a consequently greater *penetrative* action; and inasmuch as the ultimate action of iodine is that of an urgent appeal to the absorbent system for its especial activity, this is certainly a valuable consideration in the directions in which we employ it. This quality, as well as the fact of its being more concentrated than the tincture, adds to its value as a rubefacient, or as a mild counter-irritant. It cannot but be patent, moreover, that for abscessed conditions it should be superior to the tincture, because of the instant coagulation by the alcohol in the latter of the albuminoid constituents of the pus, particles of dead pulp, blood, etc., remaining in the deeper portions of the canals, or weeping into them during the operation of dressing, whereby, instead of cleansing the pulp canals and the tubuli of their contents, as we are earnestly desirous of doing, we are in reality filling them up with undissolvable particles of coagulated albumen, to be possibly pushed through the foramen at the next dressing, to perpetuate, or, at the least, prolong, an abscess, if there be one in active operation, or to develop one, if it be still undeveloped. (It may be well to say here that I do not wish to be understood as counseling the employment *inside the tooth* of *any* preparation of iodine; but in the case of its *being* used, suggest this preparation of it as the one least likely to cause after regret.) In view of these considerations, and the additional very serious one consequent upon them—viz., that if these albuminoid matters be fixed by coagulation, especially in the tubuli—all subsequent attempts to change for the better the color of the tooth, short of actual cutting out, must prove practically fruitless—it has long been my conviction that alcohols in any form—including, very especially, the *quasi* alcohols, carbolic acid and creosote—should never be allowed to *touch* the pulp canals or their contents, until by thorough and persistent cleansing with warm water, alternated with the use of the absorbent paper needles mentioned at the beginning of this article, the canals and tubuli shall have been as thoroughly cleared and freed from foreign matters as it is possible to make them by these means—nor even *then*, if, on the one hand, the tooth be dark-colored, and require blanching treatment afterwards, or, on the other, if pus or blood can weep into the canal from an unfistulated abscess. In fine, I consider the above remedies, *per se*, excellent weapons with which to war with "pus conditions" but *I do not believe the pulp canals to be a fitting place for such encounter.*

My method has long been, upon opening a dead tooth, and after cleansing as thoroughly as possible with water, broaches, paper needles, etc., to give it a two days' dressing with "Dental Pepsine." It may be my fancy, but it has always seemed to me that I accomplished thereby a very perfect *solution* of all the albuminoid matters—not only such as are contained in the canal proper, but of those in the *tubuli* as well—after which, alternate rinsings and dryings would leave the interior very pure and clean, and ready for treatment (if necessary) with aqueous solution of salicylic acid.

9. The property possessed by creosote and carbolic acid, of promptly co-agulating albumen, though in my judgment so serious a bar to their employment in pulp canals (I hope no one will understand me as therefore meaning, in *abscesses*, for they unquestionably stand foremost on the list of remedies for pus-producing surfaces, only *keep them out of the nerve canals*, except as a *dernier resort*,) render them especially valuable for lining cavities before introducing the gold, and in this I most decidedly take issue with those who make the sweeping assertion that they are of no possible value in such employment, or that they may produce such irritation of the pulp as to occasion its death. Of the former hypothesis I will speak in a moment; as to the latter, for very many years of exclusively "operative" practice I have pursued this method in practically every cavity, and have yet to see the first dead pulp which, by *any stretch of imagination*, could be ascribed to it; and it seems to me that to call dangerous to the pulp an agent that, in so continuous employment, as in every case for years has never yet had such untoward effect, savors much more strongly of the *transcendental* than the *residental.* With the questions of their value for the reduction of the sensibility of dentine previous to excavating, or for the arrest of decay under a stopping by "hardening" it or for "destroying the bacteria," or what not, I have nothing to do. I do not use them for any such purposes. For sensitive dentine I have more faith in a keen excavator, and a skillful and rapid hand, that makes every cut one of value, than I have in *any* medication—except such, indeed, as is applied simply to neutralize acid conditions, or unless temporary stoppings may be so denominated; indeed, it has always seemed to me that these applications, besides having very much of the nature of "puttering," were, at their best, but the *substitution of one hurt for another.* As to their being a specific against decay it seems unquestionable, to my mind, that if decay crops out at the margins of even perfect fillings, it will surely afterward proceed, whether saturated with creosote or not; whereas, if left in the bottom of a cavity and *hermetically* sealed with a stopping, it seems equally certain that it will *not* afterward proceed, whether saturated with creosote or not.

The use to which I put creosote and carbolic acid in a cavity, is to produce such a condition as shall lessen the effects of thermal changes after the gold shall have been introduced. With the cavity perfectly dry after excavating, I place in it a drop of either of these agents to remain two or three minutes, while I turn to prepare the gold intended for its reception. When the slight hurt of the application *has ceased*, not before, I proceed to dry and fill the cavity. I feel that I have thus coagulated the contents of the dentinal tubuli for a trifling depth, and so secured a non-conducting head—so to speak—to the contents of every tubule which leads from the filling to the pulp, and made, thereby, a more perfect non-conducting lining to every part of the cavity than could be secured by any other means, and that, too, without using up my cavity depth or holding points, as would have been done by a more tangible capping. I *know* that I have done present good by the fact of the drying out *after* the application being painless, as also will be the packing of the gold; whereas, the mere drying out *before* the application was exceedingly painful. The produced condition is not an *absolutely* non-conducting one, truly; but is sufficiently so for the great comfort of the patient; and it also tends, by greatly diminishing the shocks of thermal changes, to assist Dame Nature (by the negative process of removing obstacles so far as may be,) to reassert herself and restore the parts to as nearly normal a condition as would be possible under a gold stopping. I may here instance an item of personal experience which may be of interest in this connection: Some ten or twelve years since I had three gold stoppings inserted in as many of my inferior molars, and by three different operators; the cavities were extraordinarily alike in every particular, being very large, but shallow, and it seemed to me, with a living *soul* in each. Two were lined with creosote at my request, and I have never since particularly noticed that they existed; but in regard to the third, the gentleman who filled it declared so strongly that my suggestion was a mere fancy (of Dr. Atkinson's, I think he said,) was all nonsense, etc., that, being many years his junior, I deferred to his age, and did not insist upon its application; the event, however, proved him to have been entirely incorrect in the matter, as I was obliged for years to "engineer" all hot and cold substances past and around that tooth, because of its great sensitiveness to them, and endured much sorrow and tribulation when I accidentally or carelessly "lapse" during that time.

10. I have always been at a loss to account for the very extensive employment of myrrh which obtains for tooth powders and mouth washes and *as* a mouth wash, in the form of "tinct. of myrrh." Being a resin, and dissolved in alcohol, it is precipitated instantly it comes into contact with the fluids of the mouth, and the fine insoluble precipitate collects about the necks of the teeth, insinuating itself under the edges of the gums, making great havoc with them, and eventually with the teeth—such harm, many times, outweighing all the healing qualities that may be possessed by itself and the alcohol besides. In my own practice it is tabooed, precisely as charcoal would be, it being, to my mind, as entirely inadmissible for any employment in the mouth as the charcoal and for the same principal reason, its total insolubility in any fluids resident in the buccal cavity. It seems hardly necessary to speak of its use in tooth powders—the gum is deliberately put into *such* already precipitated!

11. For temporary dressings or trial stoppings in abscessed teeth, or in the cases frequently occurring where it is deemed expedient to postpone to the next sitting the permanent stopping of a very large cavity, I am accustomed to filling the cavity nearly full with cotton, and placing over it a facing of Hill's stopping. All-sufficient hold for the temporary requirements of the stopping may be easily obtained at the sides. This produces as perfect a cavity sealing, for the time being, as solid Hill's stopping—a quality which neither cotton, saturated with the untidy *sandarach* or the fleeting *wax*, can be depended upon to possess. It is also very easily and quickly removed; in which respect it is far superior to either the *solid* gutta-percha or sandarach on cotton. Indeed there will no doubt recur, to many who have used this latter, instances in which it has required all their skill and ingenuity to remove the large hard mass—though it was loose in the cavity—without breaking out the side walls of the tooth.

12. Whenever blood shall have gotten into a cavity after its preparation it should be *washed* out before the introduction of the gold, with *e. g.*, a little pellet of spunk, saturated with warm water; to simply dry it out will surely occasion after regret; as, although this may remove the present appearance of discoloration, the oxidation of the slight amount of blood left will, after a time, show a dark stain through the enamel, giving to the whole much the appearance of a leaky stopping. Much less should tannin be used to stop the bleeding, as this acid is one of the mordants, and sets the coloring matters of the blood; in fact, forming, with the iron in the latter, tannate of iron (ink) a stain not easily eradicated.

13. I deem it prudent, in my own practice, to *always* look carefully at the rubber dam immediately upon its removal after an operation, to be certain that it is *all there*, as, however much it may have been torn or punctured, its resilience is such as that, if held or laid flat, all torn parts will readjust themselves to their proper positions in respect to each other, and the fact of a missing piece, however, small, be readily known; as also, of course, by nothing its place on the dam, where to look for it in the mouth. And although a patient will usually point out the fact of a piece having been left between the teeth, if such *be* the case, it is obviously not so *certain* a dependence as to examine the dam—and I have known such inadvertently-left bits of rubber to accomplish serious harm.

14. I have to suggest a method which I sometimes employ with Hill's stopping, that may be of value to those who entertain the belief that this material cannot be so applied as to exclude moisture. It is simply to saturate the *perfectly* dried walls of the cavity with chloroform, and, while still wet, to paint them with a thin solution of Hill's stopping in chloroform, evaporating the latter perfectly, and packing the cavity carefully with warmed pieces of the stopping. I have long used this method, and with very excellent results, though my employment of it has been especially directed to very sensitive cavities, my object being to save the exquisitely sensitive surface the shock of the heated stopping applied *directly* to it, and also to secure the *positive* obtunding effects of the chloroform. I wish to say just here that I most decidedly antagonize the proposition recently so *absolutely* advanced, that gutta-percha stoppings *cannot* be so inserted as that they will not leak! That they usually *are* not is another hypothesis, and a possibly admissible one, inasmuch as the usual and recommended mode of insertion is to heat a single piece of the material and crowd it into the cavity, crowding out the excess, moulding it while warm, so that, thrusting it in at one side of the cavity forces it correspondingly out at the other—a course that can be fully depended upon to effectually destroy any proper adaptation to the walls—and finally completing the delicate (?) operation by trimming it, while still soft and yielding, with a thick instrument, warmed only sufficiently to drag the yielding mass back and forth as it is trimmed, and so pull it away from the walls and edges, even if it had before been perfectly adapted to them. *Per contra*, the cavity having been made *very* dry, a piece, well warmed, should be placed in one side of the cavity, condensed properly into *that* place, *held against any movement after it is once so adapted*, and care taken that no such extra pressure be brought to bear upon any new pellet, as it is added, as shall move from their *packed adaptation* the portions which have gone before. In most cavities, and with proper care in packing, there need be no necessity for trimming, as there will be no excess, and it will only remain to carefully burnish the surface smooth. If, however, there be such necessity, the stopping should be cooled, and then trimmed with a thin, burnished-surface instrument, made so hot that it shall pass over the surface and cut off the excess, without heating the body of the stopping; if more than one cut be necessary, being *very* certain that none has remained upon the instrument to ruin the whole by dragging the surface, and then, if needful, carefully making certain of the extreme edges by means of a round cold burnisher.

I firmly and determinedly believe that such a stopping will *perfectly* exclude moisture, *all* assertions to the contrary notwithstanding, and such belief is made reasonable certainty by the fact that, in the removal of such stoppings, the material does not leave the walls easily, and I am oftentimes somewhat annoyed by being obliged to "whittle" the walls clear of the layer that is in juxtaposition to them, so perfect is the adhesion.

15. In placing large amalgam stoppings, the tendency of the mass to contract in hardening, or, what would produce the same effect in a stopping, its inclination toward the assumption of a globular form, so tending to pull the material to a greater or less extent away from perfect contact with the walls and edges, I combat, and I think render almost *nil* in one of two ways, using ether, according to its applicability to the cavity to be filled, and of course *superadded* to all usual precautions, as employing the material dry, etc. One is to fill only next the walls at the first sitting, leaving the centre free, and to be filled with *any* easily removable material which can be depended upon to retain its form *inflexibly*, until the edges shall have perfectly hardened, it then being removed and its place supplied with amalgam, thereby completing the insertion, the polishing to be done afterwards. The other is to supply as much as possible of the *body* of the stopping in *one single piece*, with a cake of the same *already hardened*, (*i.e.*, from a previous using or otherwise,) the effect of which is at once seen to be that, in consequence of the great central bulk of the stopping having been already "contracted" or "globularized,"

or what not, security is had against those predilections as to the *mass* of the stopping, while as to the new material at the edges the security comes from the fact that the whole central part is hard and unyielding, and the entire amount of pulling away must consequently be confined to the shrinkage upon itself of this strip around the edges—a matter practically of no moment. My method, as to details is first to pack the sides and floor of the cavity, then to rub a slight coating of mercury over the back of the hard piece which is to occupy the centre, pack a little thickness of the new material upon this, shape its " bed " in the cavity to correspond, and then carefully settle it into its place, after which the edges may be proceeded with. The results of this practice with me have been *exceedingly* gratifying.

16. There is scarcely a question that the two especially prolific causes, among reputable operators, of failure in gold stoppings (speaking, of course, of controllable failures, and not of the inroads of disease, nor of the destruction caused by careless or slovenly patients) are *imperfect fillings at the cervical margins of approximal cavities*, and *the leaving, or making of sharp angles in the formation of cavities* in general, to be, at the best, but imperfectly filled and to afterward form centres for new decay. This bit of wise counsel was given me by one of my preceptors many years ago, when I was just beginning to creep, professionally: " My boy, give all the moments you can spare during an operation to cervical edges ; you'll never find that you've spent unnecessary time upon them." This was long years before the advent of the rubber, and when, in the stopping of approximal cavities, it was necessary to employ *every moment* to advantage. My good old friend, Dr. George E. Hawes, used to say, " Let me examine the cervical margin of a filling and I don't want to see the rest." I feel that the two maxims can scarcely be too often urged and *reiterated:* " Round the angles of your cavities," that they may be perfectly filled, and, " Pay *diligent court* to cervical margins," that their neutrality at least may be counted on, if not their friendship, in our battle with decay and disease, that they shall not *entertain* our foes if they do not turn them away; and of all things do not, by slight and neglect, leave the gates of our defenses wide open and unguarded to the very citadel, for the unopposed pouring thereinto of our most destroying enemies.—*Dental & Oral Science Magazine.*

